# Components of metabolic syndrome in relation to plasma levels of retinol binding protein 4 (RBP4) in a cohort of people aged 65 years and older

**DOI:** 10.1007/s40618-018-0856-6

**Published:** 2018-03-09

**Authors:** M. Majerczyk, P. Kocełak, P. Choręza, H. Arabzada, A. J. Owczarek, M. Bożentowicz-Wikarek, A. Brzozowska, A. Szybalska, M. Puzianowska-Kuźnicka, T. Grodzicki, A. Więcek, M. Olszanecka-Glinianowicz, J. Chudek

**Affiliations:** 1 Pathophysiology Unit, Department of Pathophysiology, Medical Faculty in Katowice, Medical University of Silesia, Medyków Street 18, 40-752 Katowice, Poland; 2 Department of Cardiology, District Hospital in Zakopane, Zakopane, Poland; 3 Health Promotion and Obesity Management Unit, Department of Pathophysiology, Medical Faculty in Katowice, Medical University of Silesia, Katowice, Poland; 4 Department of Statistics, School of Pharmacy with the Division of Laboratory Medicine in Sosnowiec, Medical University of Silesia, Katowice, Poland; 5 International Institute of Molecular and Cell Biology, Warsaw, Poland; 6 Department of Human Epigenetics, Mossakowski Medical Research Centre, PAS, Warsaw, Poland; 7 Department of Geriatrics and Gerontology, Medical Centre of Postgraduate Education, Warsaw, Poland; 8 Department of Internal Medicine and Gerontology, Jagiellonian University Medical College, Krakow, Poland; 9 Department of Nephrology, Transplantation and Internal Medicine, Medical University of Silesia, Katowice, Poland; 10 Department of Internal Medicine and Oncological Chemotherapy, Medical Faculty in Katowice, Medical University of Silesia, Katowice, Poland

**Keywords:** RBP4, Metabolic syndrome (MS), MS components, Hypertension, Triglycerides, HDL

## Abstract

**Purpose:**

Elevated plasma concentration of retinol binding protein 4 (RBP4) has recently emerged as a potential risk factor as a component of developing metabolic syndrome (MS). Therefore, this study aimed to analyse the relationship between components of MS and concentrations of plasma RBP4 in a population of subjects 65 years and older.

**Methods:**

The study sample consisted of 3038 (1591 male) participants of the PolSenior study, aged 65 years and older. Serum lipid profile, concentrations of RBP4, glucose, insulin, C-reactive protein, IL-6, and activity of aminotransferases were measured. Nutritional status (BMI/waist circumference) and treatment with statins and fibrates were evaluated. Glomerular filtration rate (eGFR), de Ritis ratio, and fatty liver index (FLI), as well as HOMA-IR were calculated.

**Results:**

Our study revealed a strong relationship between components of MS and RBP4 in both sexes: plasma RBP4 levels were increased in men by at least 3×, and in women by at least 4×. Hypertriglyceridemia was most strongly associated with elevated plasma RBP4 levels. Multivariate, sex-adjusted regression analysis demonstrated that chronic kidney disease [OR 1.86 (95% CI 1.78–1.94)], hypertriglyceridemia [OR 1.52 (1.24–1.87)], hypertension [OR 1.15 (1.12–1.19)], low serum HDL cholesterol [OR 0.94 (0.92–0.97)], and age > 80 years [OR 0.86 (0.81–0.90)] were each independently associated with RBP4 concentration (all *p* < 0.001).

**Conclusions:**

In Caucasians 65 years and older, RBP4 serum levels are associated with a number of components of MS, independent of sex and kidney function. Hypertriglyceridemia as a component of MS is most significantly related to RBP4 concentration.

## Introduction

Metabolic syndrome (MS) is a set of abnormalities which include visceral obesity, carbohydrate metabolism disturbances or type 2 diabetes, hypertriglyceridemia, decreased HDL cholesterol levels, and elevated blood pressure, [1–5]. Numerous studies have shown that the occurrence of components of MS significantly increases the risk of type 2 diabetes, cardiovascular diseases, and mortality in middle-aged populations [6–10].

Insulin resistance related to visceral obesity is the major pathway in the pathogenesis of MS [1–5]. Increased volume of visceral adipocytes is associated with local inflammation, followed by changes in adipokine release by visceral adipose tissue and low grade systemic inflammation. Both endocrine dysfunction of adipose tissue and systemic inflammation promote development of insulin resistance.

It has been shown that retinol binding protein 4 (RBP4) is one of the adipokines that participates in the development of insulin resistance by impairing insulin signalling at both the receptor and post-receptor levels, as well as by stimulation of liver gluconeogenesis [11]. The primary sources of RBP4 in vivo are hepatocytes and adipocytes [12]. Higher plasma RBP4 levels were reported in insulin-resistant humans and mice [11, 13] as well as patients with type 2 diabetes [11, 14], hypertriglyceridemia [15], atherogenic dyslipidaemia [16, 17], and hypertension [18–20].

Plasma RBP4 level correlates with abdominal fat volume [21], waist circumference, BMI, and WHR [22, 23]. Stefan et al. [24] demonstrated that RBP4 level is mainly associated with the amount of fat in the liver and presumably with hepatic insulin resistance. In addition, a stronger correlation was observed between RBP4 levels and HOMA-IR values than that between RBP4 and waist circumference [14]. Finally, RBP4 level has been considered by some researchers to be a predictor of MS development in children [25, 26] and adults, and may be a new target for MS therapy [27].

The association between plasma RBP4 levels and BMI, HOMA-IR, or other components of MS in obese patients is questioned by some researchers [15], while other researchers demonstrate correlation with BMI only in crude comparison and not in models adjusted for age and sex (for example: the Third Generation Framingham Heart Study cohort [28]). In addition, some authors have shown a strong association between circulating RBP4 levels and the number of MS components [29, 30], while others indicated that age influences the association between RBP4 and MS components [31].

So far, no studies have assessed the relationship between RBP4 levels and components of MS in older adults. Hence, the aim of this study was to evaluate if there is a relationship between circulating RBP4 levels and components of MS in a large (65+), population-based Polish cohort.

## Methods

### Study design and setting

The PolSenior study, performed in the years 2007–2012, recruited six, similarly sized, age cohorts (65–69, 70–74, 75–79, 80–84, 85–89, 90 years and older). During three visits performed by specially trained nurses, a questionnaire survey, comprehensive geriatric assessment, and measurements of body mass, height, waist circumference, and blood pressure were performed [32]. The current sub-study was based on data of 3038 available plasma samples stored at − 70 °C that were obtained from all project participants 65 years old and above in the morning after an overnight fast.

### Measurements

Height and body mass were measured with the subjects wearing light clothes and no shoes (Tanita scale BC-536, Tokyo, Japan) with an accuracy of 0.5 cm and 0.1 kg, respectively. The body mass index (BMI) was calculated according to the standard formula.

Waist circumference was measured midway between the last rib and the iliac crest in a standing position with the anterior axillary line guiding the tape-measure near the umbilicus with an accuracy of 0.5 cm.

Blood pressure (BP) measurement was performed on the right upper arm, three times, in the sitting position after a 5-min rest using a fully automatic oscillometric BP measuring device accurate to 1 mm Hg (A&D Medical, Tokyo, Japan), with the cuff selected according to the arm circumference. Thirty min before BP readings, the subjects did not smoke cigarettes, drink coffee, or engage in physical exercise [33]. Mean value was calculated from two BP measurements.

### Laboratory parameters

Plasma RBP4 concentrations were measured by ELISA (Immundiagnostik AG, Bensheim, Germany) with a 0.9-pg/mL limit of sensitivity and with mean intra- and inter-assay coefficients of < 5.0 and < 9.8%, respectively. The standard range in adults reported by the manufacturer is 20–75 µg/mL.

Serum insulin concentration was assessed by electrochemiluminescence immunoassay (ECLIA) using commercially available kits on a Cobas E411 analyser (Roche Diagnostics GmbH, Mannheim, Germany) with an inter-assay coefficient of variability of < 3.8%. Plasma interleukin-6 (IL-6) was measured by ELISA (R&D Systems, Minneapolis, MN, USA) with a sensitivity of 0.04 pg/mL and mean intra-assay and inter-assay coefficient of variance < 7.8 and < 7.2%, respectively.

Serum total cholesterol, LDL cholesterol, HDL cholesterol, triglycerides, glucose, albumin, creatinine, and C-reactive protein concentrations, and activity of alanine (ALT), aspartate (AST) transaminases, and gamma-glutamyl transpeptidase (GGT) were previously assessed by an automated system (Modular PPE, Roche Diagnostics GmbH, Mannheim, Germany) in a single certified laboratory with inter-assay coefficients of variability below 1.7, 1.2, 1.3, 1.8, 1.7, 1.7, 2.3, 5.7, 4.4, 3.2, and 1.4%, respectively.

### Data analysis

Components of MS were diagnosed according to the IDF modified criteria [34]. Any three of the following components present in an individual warranted the diagnosis of MS: 1. visceral obesity for Europeans (waist circumference ≥ 94 cm in men and ≥ 80 cm in women), 2. serum triglyceride level ≥ 1.7 mmol/L or treatment of hypertriglyceridemia, 3. low serum HDL-cholesterol concentration < 1.03 mmol/L in men and < 1.29 mmol/L in women or treatment of this condition, 4. systolic blood pressure ≥ 130 mm Hg or/and diastolic blood pressure ≥ 85 mm Hg or previously diagnosed hypertension, 5. fasting plasma glucose ≥ 5.6 mmol/L or previously diagnosed type 2 diabetes.

Insulin resistance was assessed by HOMA-IR calculated with a standard formula. The de Ritis ratio (AST/ALT) was calculated and values greater than 1 were considered to be a surrogate marker of liver damage. Fatty Liver Index (FLI) was calculated according to the equation derived from the population of the Dionysos Nutrition & Liver Study [35]; a score of 60 points or greater denoted hepatic steatosis. Glomerular filtration rate (eGFR) was estimated according to the MDRD formula [36].

### Statistical analysis

Statistical analysis was performed using STATISTICA 10.0 PL (StatSoft, Tulsa, OK, US) and StataSE 12.0 (StataCorp LLC, College Station, TX, US). Statistical significance was set at a *p* value below 0.05. All tests were two-tailed. Nominal and ordinal data were expressed as percentages, while interval data were expressed as the mean value ± standard deviation in the case of a normal distribution or as median (lower quartile–upper quartile) in the case of data with skewed or non-normal distribution. Distribution of variables was evaluated by the Shapiro–Wilk test and quantile–quantile (QQ) plots. The Levene test assessed homogeneity of variances. To evaluate the relationship between variables and RBP4 serum levels in three groups according to tertiles, the one-way ANOVA with Dunnett’s post hoc test and two-way ANOVA (metabolic syndrome components and sex) with contrast analysis were used. The stepwise backwards multivariate ordinal logistic regression was used to assess factors influencing RPB4 serum levels. Comparison of data in ordinal and nominal scale was made either with the χ^2^ test or χ^2^ trend test.

## Results

### RBP4 serum levels

Similar plasma RBP4 levels were found in men and women (medians: 41.3 vs. 41.0 ng/mL, *p* = 0.96). Therefore, lower and upper tertiles were calculated jointly for men and women after logarithmic conversion because of heavy-skewed RBP4 distribution (below 33, between 33 and 51, and over 51 ng/mL). Regardless of similar RBP4 values, but due to the differentiated distribution of components of MS between men and women, univariate analyses were done separately for men and women.

### Factors affecting plasma RBP4 concentration

In the RBP4 tertile groups (Table 1), significant differences in the occurrence of hypertension and chronic kidney disease (CKD), serum triglyceride levels, GGT activity, FLI, and eGFR values were found in both men and women. Additionally, statistically relevant differences in diastolic blood pressure, type 2 diabetes occurrence, serum glucose level, and the de Ritis ratio > 1 were noted between tertile groups only in women. Differences found only in men included the usage of fibrates (highest in the upper tertile) and serum levels of total and LDL cholesterol.

**Table 1:** Results of one-way ANOVA analysis separate for men and women in three groups according to RBP4 serum level tertiles

RBP4 tertile	Women				Men			
	Lower *n* = 483	Middle *n* = 486	Upper *n* = 478	*P*	Lower *n* = 530	Middle *n* = 527	Upper *n* = 534	*P*
Age, years	78 ± 9	78 ± 9	77 ± 8	0.49	79 ± 8	79 ± 9	78 ± 8	0.12
Age ≥ 80 years, *n* (%)	186 (38.5)	171 (35.2)	178 (37.2)	0.56	228 (43.0)	228 (43.3)	208 (38.9)	0.28
BMI (kg/m^2^)	28.7 ± 5.7	28.9 ± 5.2	29.5 ± 5.5	0.10	27.3 ± 4.4	27.1 ± 4.2	27.6 ± 4.8	0.31
Obesity, *n* (%)	186 (38.5)	193 (39.7)	202 (42.3)	0.48	130 (24.5)	126 (23.9)	143 (26.8)	0.52
Waist circumference (cm)	96.2 ± 13.5	96.1 ± 13.3	97.1 ± 13.4	0.45	100.0 ± 12.7	100.2 ± 12.9	101.4 ± 12.5	0.17
Systolic BP (mmHg)	144.6 ± 21.4	147.5 ± 21.2	145.6 ± 22.0	0.10	143.2 ± 20.1	144.5 ± 23.0	146.3 ± 22.5	0.07
Diastolic BP (mmHg)	84.2 ± 10.6	86.2 ± 10.8^*^	85.0 ± 11.1	**<** **0.05**	80.6 ± 11.0	81.5 ± 11.6	81.6 ± 11.6	0.28
Hypertension, *n* (%)	360 (74.5)	379 (78.0)	390 (81.6)**	**<** **0.01**^**$**^	344 (64.9)	364 (69.1)	391 (73.2)**	**<** **0.01**^**$**^
Type 2 diabetes, *n* (%)	106 (21.9)	115 (23.7)	139 (29.1)**	**<** **0.01**^**$**^	107 (20.2)	119 (22.6)	111 (20.8)	0.61
Serum glucose (mmol/L)	93.8 (84.5–105.7)	95.1 (86.3–107.0)	96.8 (86.9–110.0)*	**<** **0.05**	94.7 (86.5–106.4)	96.0 (86.5–109.3)	96.1 (87.3–107.4)	0.39
Insulin, µIU/mL	12.0 (8.4–17.8)	11.7 (8.1–17.4)	13.0 (8.6–18.9)	0.08	9.8 (6.6–15.1)	10.7 (6.9–15.8)	10.8 (7.3–16.1)	0.09
HOMA-IR	2.82 (1.83–4.22)	2.80 (1.74–4.51)	3.10 (1.90–4.78)	0.61	2.34 (1.41–3.79)	2.50 (1.53–4.25)	2.60 (1.59–4.10)	0.10
HOMA-IR ≥ 2.5, *n* (%)	286 (59.2)	274 (56.4)	297 (62.1)	0.19	245 (46.2)	261 (49.5)	274 (51.3)	0.24
ALT, U/L	19.7 (16.1–23.4)	19.7 (16.6–23.3)	19.0 (16.0–23.0)	0.55	19.5 (16.1–23.0)	19.9 (16.6–24.0)	20.2 (17.2–24.0)	0.15
AST, U/L	9.7 (7.2–13.1)	10.0 (7.6–13.4)	9.9 (7.4–13.1)	0.55	10.2 (7.8–13.8)	10.9 (8.0–14.8)	10.8 (8.0–14.5)	0.15
GGT, U/L	16.6 (11.2–25.5)	16.0 (11.7–25.0)	18.0* (12.7–26.6)	**<** **0.05**	18.6 (13.3–29.0)	21.0 (14.5–31.1)	22.4^#^ (15.8–35.0)	**<** **0.001**
de Ritis ratio	0.51 (0.39–0.66)	0.52 (0.39 –0.66)	0.51 (0.40–0.67)	0.71	0.54 (0.41–0.67)	0.54 (0.40–0.71)	0.54 (0.41–0.69)	0.79
de Ritis > 1, *n* (%)	14 (2.9)	19 (3.9)	31 (6.5)**	**<** **0.05**	23 (4.3)	30 (5.7)	30 (5.6)	0.54
Fatty liver index (FLI)	49.1 ± 28.8	50.4 ± 29.1	54.6 ± 29.2**	**<** **0.01**	48.5 ± 27.9	49.9 ± 27.2	54.6 ± 27.8^#^	**<** **0.001**
FLI > 60, *n* (%)	185 (38.3)	201 (41.4)	223 (46.7)**	**<** **0.05**	201 (38.0)	194 (36.9)	237 (44.4)^**^	**<** **0.05**
Total cholesterol, mmol/L	211.0 ± 45.6	213.9 ± 46.7	211.4 ± 51.8	0.59	187.4 ± 39.8	198.4 ± 45.1^#^	196.6 ± 44.4^**^	**<** **0.001**
LDL cholesterol, mmol/L	125.0 ± 40.4	126.5 ± 39.6	123.6 ± 43.9	0.54	111.9 ± 35.7	120.7 ± 39.0^#^	115.4 ± 37.8	**<** **0.001**
HDL cholesterol, mmol/L	53.0 ± 13.8	53.9 ± 14.1	53.3 ± 14.8	0.58	48.2 ± 12.6	48.7 ± 13.7	48.3 ± 13.5	0.79
Triglycerides, mmol/L	115.6 (89.2–150.0)	121.0 (91.6–161.3)	129.3^#^ (98.3–167.5)	**<** **0.001**	98.5 (75.1–131.3)	104.5^*^ (84.2–135.1)	113.6^#^ (88.7–153.9)	**<** **0.001**
Statins, *n* (%)	114 (23.6)	121 (24.9)	138 (28.9)	0.15	112 (21.1)	117 (22.2)	139 (26.0)	0.14
Fibrates, *n* (%)	7 (1.4)	3 (0.6)	9 (1.9)	0.21	2 (0.4)	7 (1.3)	12 (2.2)	**<** **0.05**
hs-CRP, mg/L	2.3 (1.1–5.0)	2.4 (1.2–4.5)	2.5 (1.3–4.6)	0.76	2.3 (1.0–5.2)	2.0 (0.9–4.9)	2.3 (1.0–5.0)	0.43
hs-CRP ≥ 3 mg/L, *n* (%)	205 (42.4)	200 (41.1)	207 (43.3)	0.79	222 (41.9)	207 (39.3)	214 (40.1)	0.67
Interleukin 6, pg/mL	2.2 (1.5–3.7)	2.2 (1.4–3.4)	2.0 (1.4–3.4)	0.34	2.6 (1.6–4.3)	2.4 (1.6–3.8)	2.2 (1.3–3.8)	0,08
eGFR_MDRD_ mL/min/1.73 m^2^	79.9 ± 20.6	75.5 ± 19.6^#^	77.3 ± 22.8^#^	**<** **0.001**	82.7 ± 20.0	77.9 ± 19.8^#^	75.1 ± 23.8^#^	**<** **0.001**
eGFR < 60 mL/min/1.73 m^2^, *n* (%)	121 (25.0)	139 (28.6)	185 (38.7)^#^	**<** **0.001**	101 (19.1%)	150 (28.5%)^**^	178 (33.3%)^#^	**<** **0.001**

Data presented as mean value ± SD or median (lower–upper quartile)

Bold values indicate statistically significant results

*BMI* body mass index, *hs-CRP* C-reactive protein, *eGFR* estimated glomerular filtration rate, *HOMA-IR* homeostatic model assessment of insulin resistance, *ALT* alanine transaminase, *AST* aspartate transaminase

^$^For trend; in comparison to the lower quartile: *p < 0.05; ** *p* < 0.01; ^#^*p* < 0.001

Multivariate stepwise ordered logistic regression analysis of factors influencing RBP4 levels revealed that chronic kidney disease had the strongest influence both in crude and sex-adjusted analyses. Other factors that positively correlated with RBP4 levels after sex adjustment were hypertriglyceridemia and a higher systolic BP. An inverse relationship was observed in the crude model for subjects in excess of 80 years of age and decreased serum HDL cholesterol concentration or its treatment. After sex adjustment, the de Ritis ratio > 1 lost statistical relevance. Likelihood of fatty liver, expressed as FLI > 60, did not correlate with RBP4 levels (Table 2).

**Table 2:** Results of multivariable stepwise ordered logistic regression analysis of factors influencing plasma RBP4 levels

Factor	Crude		Sex-adjusted	
	OR	± 95% CI	OR	± 95% CI
Female	–	–	Not considered in the model	
Age > 80 years	0.862*	0.749–0.991	0.856^#^	0.813–0.901
Visceral obesity	–	–	–	–
Serum triglycerides levels ≥ 150 mg/dL or treatment of hypertriglyceridemia	1.494^#^	1.281–1.741	1.520^#^	1.236–1.869
Serum HDL-cholesterol concentration: < 40 mg/dL in men and < 50 mg/dL in women or treatment this lipid disturbances	–	–	0.944^#^	0.920–0.967
Systolic blood pressure ≥ 130 mmHg or/and diastolic blood pressure ≥ 85 mmHg or previously diagnosed hypertension	–	–	1.152^#^	1.116–1.188
Fasting plasma glucose ≥ 100 mg/dL (≥ 5.6 mmol/L) or previously diagnosed type 2 diabetes mellitus	–	–	–	–
hs-CRP > 3 mg/L	–	–	–	–
de Ritis ratio > 1	1.392^*^	1.021–1.898	–	–
FLI: > 60	–	–	–	–
eGFR < 60 mL/min/1.73 m^2^	1.858^#^	1.573–2.195	1.861^#^	1.781–1.944

*p < 0.05; **p < 0.01; ^#^*p* < 0.001

### RBP4 and MS components

Our study revealed a strong relationship between components of MS and RBP4, both in women (*p* < 0.01) and men (*p* < 0.001). Hypertriglyceridemia (*p* < 0.001 for both sexes), occurrence of type 2 diabetes or fasting plasma glucose over 100 mg/dL (*p* < 0.01, *p* < 0.05 for women and men, respectively) were substantially related to RBP4 levels (Table 3).

**Table 3:** Comparison of MS factors presence according to RBP4 serum level tertiles, separate for men and women

RBP4 tertile	Women				Men			
	Lower *n* = 483	Middle *n* = 486	Upper *n* = 478	*P*	Lower *n* = 530	Middle *n* = 527	Upper *n* = 534	*P*
Metabolic syndrome, *n* (%)	313 (64.8)	332 (68.3)	356 (74.5)	**<** **0.01**^**$**^	258 (48.7)	282 (53.5)	323 (60.5)	**<** **0.001**^**$**^
Visceral obesity, *n* (%)	437 (90.5)	432 (88.9)	431 (90.2)	0.687	372 (70.2)	384 (72.9)	407 (76.2)	**<** **0.05**^**$**^
Serum triglycerides levels ≥ 150 mg/dL or treatment of hypertriglyceridemia, *n* (%)	128 (26.5)	149 (30.7)	178 (37.2)	**<** **0.001**^**$**^	83 (15.7)	101 (19.2)	157 (29.4)	**<** **0.001**^**$**^
Serum HDL cholesterol concentration: < 40 mg/dL in men and < 50 mg/dL in women or treatment this lipid disturbances, *n* (%)	273 (56.5)	268 (55.1)	287 (60.0)	0.286	220 (41.5)	229 (43.4)	244 (45.7)	0.387
Systolic blood pressure ≥ 130 mmHg or/and diastolic blood pressure ≥ 85 mmHg or previously diagnosed hypertension, *n* (%)	419 (86.7)	437 (89.9)	431 (90.2)	0.168	434 (81.9)	429 (81.4)	457 (85.6)	0.140
Fasting plasma glucose ≥ 100 mg/dL (≥ 5.6 mmol/L) or previously diagnosed type 2 diabetes, *n* (%)	194 (40.2)	215 (44.2)	232 (48.5)	**<** **0.01**^**$**^	210 (39.6)	251 (47.6)	240 (44.9)	**<** **0.05**
No. of metabolic syndrome factors	3.0 ± 1.2	3.1 ± 1.2	3.3 ± 1.2^**^	**<** **0.01**	2.5 ± 1.2	2.6 ± 1.2	2.8 ± 1.3^#^	**<** **0.001**

Bold values indicate statistically significant results

^$^For trend; in comparison to the lower quartile: *p < 0.05; **p < 0.01; ^#^*p* < 0.001

### RBP4 and the number of components of MS

**Fig. 1:**
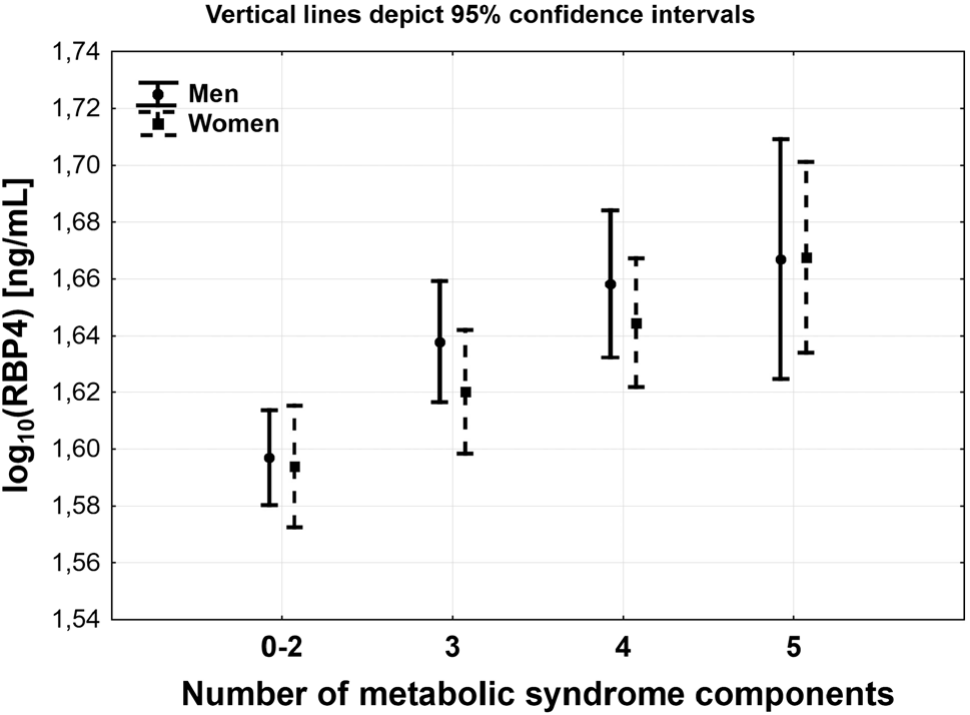
RBP4 serum levels according to the number of metabolic syndrome factors, separated for men and women

Two-way ANOVA was run to examine how the number of components of MS and sex influence the RBP4 serum levels. There was no significant interaction between the number of components of MS and sex (*p* = 0.88). Simple main effects analysis showed a significant association of the number of components of MS (*p* < 0.001) with the level of RBP4, but there were no differences between men and women (*p* = 0.39) (Fig. 1).

In comparison to the group without MS (less than three components of MS), RBP4 serum levels were higher in groups with at least three components for men and with at least four components for women (Fig. 1).

## Discussion

Our large, population-based, cohort study shows that increased RBP4 serum levels are strongly associated with the presence and the number of components of MS in an 65 + Caucasian population. These findings correspond with previous studies performed in multi-ethnic and over 65-year-old populations [37–39]. The findings support the hypothesis that RBP4 plays a role in the pathogenesis of the metabolic complications found in obesity.

In older adults the association between RBP4 levels and components of MS, especially carbohydrate disturbances, is weaker and affected by impaired renal function. The strong associations between RBP4, insulin resistance, and the percent of trunk fat in young subjects were shown to disappear in older adults [31]. However, an explanation of the phenomenon is missing.

A significant effect on RBP4 metabolism is impaired kidney function, which is frequently found in older adults. This supposition is supported by previously published data showing normalisation of RBP4 levels after kidney transplant [40–44] and by our results presenting an inverse relationship between eGFR and RBP4 levels. Unfortunately, the lack of assessment of kidney function in the numerous published studies may be a reason for the inconsistency of available data.

The most significant findings of our study are the associations between the levels of RBP4 and hypertriglyceridemia, as well as low HDL cholesterol levels (in the multivariate analysis). This suggests that RBP4 might play an important role in the disturbance of lipid synthesis in the liver, related to insulin resistance, and perhaps is a surrogate marker of liver steatosis. It was shown that RBP4 expression was aberrantly elevated in non-alcoholic fatty liver disease (NAFLD) in middle-age human and animal models, as well as positively associated with hepatic mitochondrial dysfunction combined with increased hepatic triglyceride accumulation [45, 46]. In vitro studies showed that RBP4 stimulated lipogenesis in HepG2 cells in a dose-dependent manner [47]. In line with these findings, we demonstrated a higher prevalence of subjects with increased FTI > 60 and de Ritis index > 1 in the highest RBP4 tertile. However, in multivariate regression analysis, both an increased FLI (strongly affected by triglyceride level) and the de Ritis index lost statistical significance. This suggests that RBP4 is one of many factors influencing hepatic lipogenesis and, therefore, its level is not a sensitive marker of NAFLD, at least in older adults.

A strong association between circulating levels of RBP4 and triglycerides seems to reflect the increased lipogenesis in the study subjects. This supposition is strengthened by Rocha et al., who demonstrated that on middle-aged, morbidly obese patients, RBP4 independently correlates with triglycerides, HDL, LDL, VLDL (very-low-density lipoprotein), and small-HDL subfractions of cholesterol; all particles enriched in triglycerides [17]. There are a couple of hypotheses explaining the stimulatory effect of RBP4 on lipogenesis. RBP4 has been shown to stimulate synthesis of SREBP-1 (sterol regulatory element-binding protein-1) and PPARγ (peroxisome proliferator-activated receptor gamma) in the liver, both proteins involved in lipogenesis [48]. RBP4 can also modulate lipid homeostasis indirectly, through retinoids, by activation of nuclear retinoid receptors. Finally, retinoids and retinol-binding proteins modulate apolipoprotein C-III production, an enzyme involved in the catabolism of VLDL (an inhibitor of lipoprotein lipase) and β-oxidation [49]. In a kinetic study, Verges et al. demonstrated reduced VLDL catabolism (VLDL-apolipoprotein B100 fractional catabolic rate) in diabetics with increased RBP4 levels [50].

This study has some limitations, such as the lack of validation of the utilised ELISA kit by a quantitative western blot analysis [23], liver sonography, and visceral fat depot assessment by DEXA or CT. It should be stressed that such measures are difficult to apply in population-based studies performed in their place of residence.

## Conclusions

In older adult Caucasians, RBP4 serum levels are associated with a number of components of MS, independent of sex and kidney function. Hypertriglyceridemia as a component of MS is most significantly related to RBP4 concentration.
